# A Case of *Listeria monocytogenes* Infective Endocarditis

**DOI:** 10.1155/2022/5958017

**Published:** 2022-08-08

**Authors:** Faraz Badar, Refayat Bhuiyan, Shaha Nabeel

**Affiliations:** ^1^Internal Medicine Residency Program, John T. Mather Memorial Hospital, NY, USA; ^2^Hematology Oncology Fellowship Program, Jacobi Medical Center, NY, USA

## Abstract

*Listeriamonocytogenes* (*L*. *monocytogenes*) is a Gram-positive facultative intracellular aerobic bacterium. We report the case of a 77-year-old male presenting with focal neurological symptoms found secondary to embolization from *L*. *monocytogenes* infective endocarditis (IE) involving the mitral valve. Blood cultures and echocardiogram were positive. Meningitis was ruled out via lumbar puncture. Treatment was done successfully with a prolonged course of ceftazidime. IE from *L*. *monocytogenes* is a rare but life-threatening complication and therefore prompt identification of infectious process is paramount. The mainstay of therapy is use of beta-lactams.

## 1. Introduction

*Listeria monocytogenes* (*L*. *monocytogenes*) is a Gram-positive facultative intracellular aerobic bacterium which has emerged as a prominent food-borne pathogen in recent years. A commonly implicated source is contaminated delicatessen products intended to be eaten without heating [1]. Infective endocarditis (IE) is a rare complication of this bacterial infection, and only about 8% of *L*. *monocytogenes* infections develop it [2]. Prosthetic and native valve infections have been reported, with presentations ranging from localized valve infections to systemic involvement. Due to scarcity of this disease, both diagnosis and treatment are challenging. Here, we present a case of native valve endocarditis (NVE) caused by *L*. *monocytogenes*, an uncommon pathogen in endocarditis.

## 2. Case

A 77-year-old male was brought to the emergency department for sudden onset altered mental status and right-sided facial droop with expressive aphasia. His past medical history consisted of type 1 diabetes mellitus, and a four-vessel coronary artery bypass graft with concurrent bioprosthetic aortic valve replacement (BioAVR). Physical examination was notable for expressive aphasia and mild facial droop, whereas auscultation of the heart and lungs was benign. Initial laboratory testing revealed a leukocytosis of 13,200 per microliter with a predominance of 75% polymorphonuclear cells, as well as mildly elevated troponins which trended down on serial measurement. Electrolytes, toxicology, arterial blood gas, lactate, and creatine phosphokinase were within normal limits. Initial electrocardiogram (EKG) showed atrial fibrillation with a rate of 95 beats/min. Chest X-ray and computed tomography (CT) of the head were negative for acute pathology. Patient was initiated on full dose anticoagulation with enoxaparin due to his EKG findings. Later the same day, magnetic resonance imaging (MRI) of the head was performed, which demonstrated multiple acute to subacute infarcts involving bilateral temporoparietal regions, frontal lobes, basal ganglia, right pons, and right cerebellum. Distribution was suggestive of an embolic phenomenon involving both anterior and posterior circulations. Due to these findings, vancomycin and piperacillin-tazobactam were initiated for broad-spectrum coverage. A transthoracic echocardiogram (TTE) also performed that day revealed a 9 mm mobile, rounded echodensity on the atrial aspect of the mitral leaflet. The ejection fraction was 60–65%. Preliminary blood cultures reported Gram-positive rods in aerobic and anaerobic bottles. Piperacillin-tazobactam was changed to ceftazidime 2 g IV every 8 hours for improved central nervous system (CNS) coverage. On day three of hospital stay, final cultures were identified as *Listeria monocytogenes* and ampicillin 2 g IV every 4 hours was initiated. The growth media used were blood, Columbia, chocolate, and CDC agars, whereas the identification technique was DNA PCR assay. Antimicrobial susceptibilities were unavailable due to limited laboratory protocols for *Listeria*. A lumbar puncture (LP) was also performed to rule out *Listeria monocytogenes* meningitis which did not show any leukocytes or organisms.

Over the course of this patient's hospital stay, his symptoms gradually improved; speech became more comprehendible and facial droop almost resolved. Repeat TTE 4 weeks also later revealed reduction in size of the vegetation. A peripheral intravenous central catheter (PICC) was placed, and patient was discharged on a 6-week course of ampicillin without any long-term sequelae of IE on outpatient follow-up.

## 3. Discussion

*Listeria monocytogenes* is commonly found in many foods, especially processed meats and cheeses. It is able to tolerate high salt, low pH, and even hypothermic environments. This gives *L*. *monocytogenes* the ability to withstand many of the food preservation processes and contaminate foods such as improperly heated meats and cold cuts, and it can be found in store-made salads containing chicken or seafood. *L*. *monocytogenes* is a short Gram-positive facultative intracellular coccobacillus and is a transient colonizer of the human gastrointestinal (GI) tract. Infection can occur after ingestion of a large inoculum of bacteria from contaminated foods, with a mean incubation period of 24 hours. This bacterium has a predisposition to affect immunocompromised individuals with predisposing factors. Endocarditis from this bacterium is an even more unusual finding but a life-threatening one that warrants prompt diagnosis and treatment. History of damage to cardiac valves and signs and symptoms of heart failure are frequently elicited at presentation, and almost one-third of the cases involve prosthetic valves [2].

Furthermore, a diagnosis of IE requires a high suspicion, and treatment may be delayed until certain signs are present. Sudden-onset cerebrovascular accident (CVA) in a person with history of cardiac valve repair should prompt a provider to have IE high on the list of differentials. Mortality rates are 2.4 times higher in patients with neurological manifestations [3]. *L*. *monocytogenes* IE, usually presenting in a subacute manner, may make this diagnosis even more difficult. Occurrence of *L. monocytogenes* IE is uncommon, with a mortality of 100% in untreated patients.

Due to rarity of this pathogen, there is no established optimal treatment for *L*. *monocytogenes* IE. Review of literature reveals a variety of antibiotics have been used. The most commonly used antimicrobial regimens were ampicillin or amoxicillin monotherapies, or a combination of beta-lactams with aminoglycosides such as gentamycin, to create a synergistic effect. According to a recently published review of cases of *L*. *monocytogenes* IE, medical management with beta-lactams alone showed better survival benefits in native valve endocarditis (NVE), whereas patients with prosthetic valve endocarditis (PVE) had superior outcomes with beta-lactam and aminoglycoside combination therapy [4]. Although additional research is needed on this topic, patients with a prior sensitivity to penicillin have demonstrated good response to treatment after desensitization [5]. Also, despite the prevailing notion that valve surgery is crucial for optimal management of complicated IE, data supporting this have been inconclusive [6, 7]. Previous studies have shown that antimicrobial use alone demonstrated superior survival outcomes in NVE versus a combination of medical and surgical management, which was found to have a profound survival benefit for PVE [4].

Our patient was treated with ampicillin alone. Aminoglycosides were avoided due to underlying acute kidney injury, and surgery was deferred due to high predicted mortality. He responded well to monotherapy, with complete resolution of symptoms.

## Data Availability

The case details are present in the Electronic Medical Record (EMR) system of the hospital. Previously reported data are cited at relevant places within the text as numbered references.

## Additional Points

*Summary*. Though we often think of *Listeria monocytogenes* as a food-borne pathogen or cause of bacterial meningitis, our case illustrates another rare but serious complication of infection with this organism. The mainstay of therapy is IV ampicillin. Aminoglycosides may be used for synergistic effects but can be associated with increased toxicity.
